# Unlocking the Potential of Nano-Enabled Precision Agriculture for Efficient and Sustainable Farming

**DOI:** 10.3390/plants12213744

**Published:** 2023-11-01

**Authors:** Vinod Goyal, Dolly Rani, Ritika, Shweta Mehrotra, Chaoyi Deng, Yi Wang

**Affiliations:** 1 Department of Botany and Plant Physiology, CCS Haryana Agricultural University, Hisar 125004, Haryana, India; 2 Department of Microbiology, CCS Haryana Agricultural University, Hisar 125004, Haryana, India; 3 Department of Bio and Nano Technology, Guru Jambheshwar University of Science and Technology, Hisar 125001, Haryana, India; 4 Department of Analytical Chemistry, Connecticut Agricultural Experiment Station, New Haven, CT 06511, USA; chaoyi.deng@ct.gov (C.D.); yi.wang@ct.gov (Y.W.)

**Keywords:** nanotechnology, nano-fertilizers, nano-biofertilizers, nano-particles (NPs), toxicity, microorganisms, plant growth-promoting rhizobacteria (PGPR)

## Abstract

Nanotechnology has attracted remarkable attention due to its unique features and potential uses in multiple domains. Nanotechnology is a novel strategy to boost production from agriculture along with superior efficiency, ecological security, biological safety, and monetary security. Modern farming processes increasingly rely on environmentally sustainable techniques, providing substitutes for conventional fertilizers and pesticides. The drawbacks inherent in traditional agriculture can be addressed with the implementation of nanotechnology. Nanotechnology can uplift the global economy, so it becomes essential to explore the application of nanoparticles in agriculture. In-depth descriptions of the microbial synthesis of nanoparticles, the site and mode of action of nanoparticles in living cells and plants, the synthesis of nano-fertilizers and their effects on nutrient enhancement, the alleviation of abiotic stresses and plant diseases, and the interplay of nanoparticles with the metabolic processes of both plants and microbes are featured in this review. The antimicrobial activity, ROS-induced toxicity to cells, genetic damage, and growth promotion of plants are among the most often described mechanisms of operation of nanoparticles. The size, shape, and dosage of nanoparticles determine their ability to respond. Nevertheless, the mode of action of nano-enabled agri-chemicals has not been fully elucidated. The information provided in our review paper serves as an essential viewpoint when assessing the constraints and potential applications of employing nanomaterials in place of traditional fertilizers.

## 1. Introduction

Agriculture provides the majority of the country’s livelihoods in emerging nations like India, where it supports about two-thirds of the population. India’s production of food grains has expanded fivefold since independence, reaching several million tonnes. High demand for agricultural products places significant pressure on the development of new agricultural practices since the use of high-yielding chemically synthesized fertilizers, farmyard manures, bio-manures, etc. enhances agricultural production. Chemical fertilizers have become a key component of the Indian agricultural system to meet the expanding demand, agricultural output, and nutritional quality [1]. Providing comprehensive macro- and micronutrients, which soil typically lacks, is the primary goal of fertilizer use in agriculture [2]. Although some fertilizers directly impact plant development, 35–40% of crop yield depends on fertilizer use. Agrochemical use over an extended period has had a detrimental effect on human and soil health [3]. This has been a significant contributor to soil and water pollution, soil erosion, nutrient imbalance, reduced agro-biodiversity, lower soil fertility, low water holding capacity, and disruptions in the flora and fauna of the natural soil ecosystem [4]. Application of excessive nutrients pollutes the soil, and a scarcity of water makes the problem worse [5]. Plants do not take up a large part of synthetic material but rather flow off from fields and end up in water bodies [3]. Significant worries regarding food safety and quality have been raised due to the excessive use of synthetic fertilizers [6]. Developing sustainable agriculture practices needs to be prioritized to overcome these complications. However, chemical fertilizers cannot be totally substituted, but there are a number of techniques to increase their efficiency. In order to reduce environmental stresses and boost crop productivity, it is necessary to establish eco-friendly strategies [7].

Nanotechnology may be one solution to address all of these issues more appropriately [8]. Due to their small size, increased surface area, and compatibility with flexible substrates, nano-materials (1–100 nm) are used in multiple sectors [9]. Precision agriculture utilizes nanotechnology in various ways, including the production of agricultural goods such as slow and controlled-release fertilizers, nano-herbicides, nano-fungicides, and nano-sensors for various analyses [10]. The uses of nanotechnology in the agri-food industry have been acknowledged by numerous nations to satisfy the demands of the growing population and the diminishing natural resources [11]. The potential for agriculture to improve fertilization, plant growth regulators, and pesticides may expand with the development of nano-materials in environmentally safe and sustainable ways [12]. Consequently, this technology aids in lowering environmental pollution. The vast surface area of nanoparticles makes them appealing to address problems that physical, chemical insecticides, and biological control approaches cannot. In addition to offering new agrochemical agents and delivery systems to increase crop output, nanotechnology also promises to reduce the use of pesticides [13]. Applications of nanotechnology in agriculture include the production of nano-formulations, the use of nano-sensors in crop protection for identifying diseases and agrochemical residues, and postharvest and pest management [14]. Precision farming techniques may be applied to increase agricultural yields while avoiding harm to the soil and water. Moreover, it can lessen nitrogen loss via emissions, leaching, and soil microbes [15]. Nanotechnology has several applications in all phases of the production, processing, storage, packing, and transportation of agricultural products [16]. Nanotechnology holds substantial potential for agriculture; nevertheless, certain concerns remain to be addressed as an integral aspect of risk assessment.

For the synthesis of nanoparticles, a variety of techniques can be used, although these techniques can be broadly categorized into two classes: top-down and bottom-up approaches. Top-down approaches use destructive methods, whereas bottom-up strategies use the building-up approach. Based on their operation, reaction conditions, and established procedures, these techniques are further divided into several subclasses, including physical, chemical, and biological. Physical and chemical processes used to synthesize nanoparticles are difficult, expensive, and out-of-date and produce toxic waste that is dangerous to both human and environmental health [17]. Additionally, the difficulties of scaling up the process, the separation and purification of nanoparticles from the micro-emulsion, and the high surfactant consumption make these approaches have significant drawbacks [18]. For the synthesis of nanoparticles, the biological approach is preferable to the physical and chemical approaches [19]. Many scientists are interested in green and biogenic bottom-up synthesis since it is practical and uses less hazardous techniques. These procedures are economical, simpler, quicker, safer, more environmentally friendly, and, most importantly, non-toxic. In addition, it requires far less energy, wastes less resources, and controls chemicals and reagents in a more useful way. The fact that this is a bottom-up strategy is an added benefit [20,21,22]. The use of plant extracts, bacteria, fungi, algae, and actinomycetes in the green synthesis of the nanoparticle is favorable.

Nano-enabled agriculture is an emerging concept [23]. To improve the quality attributes within them, nanotechnology is emerging as an intriguing choice in the form of nano-fertilizers and nano-biofertilizers. A nano-fertilizer consists of nutrient formulations that may be administered to plants and allow for prolonged and uniform uptake. Research has demonstrated that nano-fertilizers help plants utilize nutrients more efficiently, minimize soil toxicity, moderate the negative consequences of overuse of chemical fertilizers, and increase the rate of fertilizer application [24,25,26]. Employing nano-fertilizers significantly minimizes waste, resulting in cost savings and environmental protection. In addition, nano-fertilizers and beneficial microorganisms, called nano-biofertilizers, have established significant approaches towards sustainable agriculture. A biocompatible nanomaterial and a biological source-driven fertilizer are deliberately combined to produce a nano-biofertilizer, which has great efficiency for both components. These characteristics are intended to promote steady nutrient release over an extended period of crop growth, which will enhance nutrient consumption and increase the yield of crops and sustainability [27]. To determine the environmentally safe doses of nano-fertilizers, a thorough investigation in the field and in a lab is required [28]. In the context of agricultural innovation, nano-fertilizers and nano-biofertilizers offer great potential for boosting crop productivity. However, these novel products often face limited adoption due to reduced commercialization. Addressing this issue requires disseminating scientific evidence of their effectiveness among farmers [29]. Regulating nanotechnology-based agri-products in India involves a collaborative effort among various authorities and legal frameworks. The Department of Biotechnology (DBT), Ministry of Environment, Forest and Climate Change (MoEFCC), Food Safety and Standards Authority of India (FSSAI), and Indian Council of Agricultural Research (ICAR) play crucial roles in ensuring the safety and efficacy of these products. Under the Environment (Protection) Act of 1986, the production, import, export, and use of hazardous substances, including nano-materials, is monitored to minimize potential environmental risks. The Hazardous Waste Rules of 2016 further emphasize the responsible management of hazardous waste, including nano-materials. Ensuring food safety and quality falls under the jurisdiction of the FSSAI, which establishes guidelines and standards for agri-products, including those involving nanotechnology. Meanwhile, the Insecticides Act of 1968 regulates nanotechnology-based insecticides, ensuring thorough scrutiny and proper usage. Guidelines for nanoparticle characterization, provided by the Indian Pharmacopoeia Commission (IPC), offer a standardized approach to assessing the properties of nanoparticles used in agri-product production. This promotes the consistency and reliability of data related to these nanoparticles. India’s regulatory framework for nanotechnology-based agri-products seeks to balance the potential benefits of innovation with safety considerations. By combining scientific evidence, collaborative regulation, and standardized guidelines, we can facilitate the responsible use of nanotechnology in agriculture while safeguarding the environment and human well-being [30].

The article provides a comprehensive overview of nanotechnology in agriculture, focusing on nano-fertilizers and nano-biofertilizers, their sources, benefits, effectiveness, and potential for sustainable agriculture, as evident in the graphical representation. It addresses the role of nanoparticles in biotic and abiotic stress alleviation, soil remediation, and nutrient enhancement in agricultural crops. It emphasizes the need to balance the potential benefits of nanotechnology with biosafety considerations.

Graphical representation illustrating the types of nanoparticles and their morphological, biochemical and physiological effects on plants.

## 2. Microbial Nanoparticles

Nanoparticles in pharmacology, medicine, and agriculture contribute to their direct entry into the human body [31]. As a result, developing an environmentally acceptable and low-cost method of synthesizing nanoparticles is critical. There are numerous organisms capable of synthesizing and using nanoparticles [32,33]. Because significant nanomaterial applications are typically size-dependent, controlled synthesis of nanomaterials in size is desirable [34]. For the physical and chemical production of nanoparticles, reducing agents are required that have a strong reactivity and toxic effect for human use or are environmentally harmful and highly expensive [35]. Large-scale synthesis has a number of challenges, including low stability and mono-dispersity [36]. Utilizing microbes is one of the most prominent approaches employed in producing nanoparticles employing diverse biological methods [37]. Nanoparticles of microbial flora such as bacteria, fungi, yeast, microalgae, and viruses have been successfully synthesized and proved to be useful. Green synthesis of nanoparticles uses bacteria, fungus, algae, viruses, and plants as reducing and stabilizing agents [38]. The utilization of biological components has various advantages over traditional chemical processes, including environmental friendliness, biocompatibility, and cost-effectiveness [39].

### 2.1. Nanoparticles of Bacteria

Bacteria have developed a variety of mechanisms for tolerating heavy metals, including the transit of heavy metals across cell membranes, deposition on cell walls, intracellular and extracellular trapping, the development of complexes, and redox reactions, all of which provide the foundation for various bioremediation techniques [40]. These processes encompass the efflux system, solubility fluctuations caused by altering the redox potential of metal ions, extracellular conjunction, precipitation, and failure in the transmission of specific metal systems [41]. Additionally, bacteria strains that cannot survive in high metal concentrations can be used as useful microorganisms [42].

Bacteria have been shown to be capable of binding and concentrating dissolved metal and metalloid ions. Individual bacteria can transform hazardous metal ions into non-toxic Nanoparticles [43,44]. As a result, some bacteria are exploited as nano-factories, allowing for a novel approach to removing metal or metalloid ions and creating materials with distinct features [45]. Bacteria are particularly essential tools for obtaining Nanoparticles among “green” synthesis methods due to their diversity and excellent adaptation to severe environments [46]. Because of its low energy consumption and process controllability, bacterial nanoparticles synthesis is particularly promising. Nanoparticles can be synthesized both intracellularly and extracellularly by various bacteria. Positively charged metal ions are attracted to the negatively charged bacterial cell membrane during intracellular production due to electrostatic contact. Furthermore, the bacterial cell membrane has enzymes that decrease metal ions to the nano-scale [31]. Bacteria and actinomycetes have been found to be the best candidates for nanoparticle synthesis [47]. Some bacterial species can use specialized defense mechanisms to combat stressors such as heavy metal ion toxicity. Some species, such as *Pseudomonas stutzeri* and *Pseudomonas aeruginosa*, have been demonstrated to survive and proliferate in high metal ion concentrations [48]. *Bacillus* sp., isolated from the medicinal plants, *Adhatoda beddomei* (malabar nut) and *Garcinia xanthochymus* (egg tree), could reduce silver nitrate to produce Ag NPs [47]. Bacterial synthesis of Ag NPs involves biomass, supernatant, cell-free extract, and derived components. Extracellular synthesis is preferred due to easy recovery. Bacteria play significant roles in Ag NP synthesis, with silver-resistant genes, c-type cytochromes, peptides, and cellular enzymes playing crucial roles. Organic materials control nanoparticle morphology, dispersion, and yield. Ag NPs have antioxidant and antimicrobial properties, making them promising as nanoantibiotics [49]. Alam and his co-workers synthesized nontoxic selenium nanoparticles, which showed antibacterial activity against five strains, inhibiting and degrading bacterial biofilms [50]. The study by Dong and his co-workers established the antibacterial activity of silver nanoparticles synthesized from the endophytic strain SYSU 333,150 against *S. warneri* [51]. A bacterial strain (*Pseudomonas stutzari* AG259) isolated from silver mines could produce silver nanoparticles extracellularly [52]. *Desulfovibrio desulfuricans*, sulfate-reducing bacteria, could produce palladium nanoparticles [53].

In general, the metabolic process is thought to be responsible for nanoparticles bioreduction in living bacteria. However, several investigations have revealed that active bacteria and even dead versions of particular bacteria can be employed for nanoparticles production. The mechanisms of these processes, however, may differ.

### 2.2. Nanoparticles of Cyanobacteria

Cynobacteria are significant agricultural and food microorganisms. They are also employed as biofertilizers or organic fertilizers, which contain live organisms and utilize naturally available inputs such as solar energy, macronutrients, and water to assure soil fertility and plant growth. Small-scale farmers that use biofertilizers have the potential to produce greater, more sustainable yields as well as healthier soils for themselves and their communities [54]. In phyco-nanotechnology, the algal extract is employed to generate nanoparticles. Algal extracts are employed because they are less toxic, easier to work with, and can grow at low temperatures. Algal phytochemicals act as an efficient metal-reducing and capping agent in one-step synthesis to generate a lasting coating on metal nanoparticles [55]. Priyadharshini et al. [56] synthesized ZnO NPs using an aqueous extract of the macroalga *Gracilaria edulis*. Since algae can make nanoparticles from living and dead materials, they are commonly called bio-nanomaterials. The most commonly used algae for producing metallic nanoparticles include members of the Chlorophyceae, Phaeophyceae, Cyanophyceae, Rhodophyceae, and several kinds of diatoms [57]. Nanoparticles produced by microorganisms have biological uses in the disciplines of bioremediation, biomineralization, bioleaching, and biocorrosion [42].

### 2.3. Nanoparticles of Fungi

The use of fungi in the production of nanoparticles has also been recognized. Myco-nanotechnology has a high demand and enormous potential, partly because of the diversity and vast range of fungi [58,59]. The fungal proteins have the ability to reduce and hydrolyze metal ions. Fungi are also simple to isolate and cultivate. Fungi can produce metal nanoparticles both intracellularly and extracellularly [60]. Fungi may collect metal ions through physical, chemical, and biological processes, such as extracellular attachment by metabolites and certain polypeptides [61]. Extracellular synthesis is more efficient and convenient for nanoparticle extraction. In this situation, biosynthetic metal nanoparticles are more resistant to oxidation, allowing them to be used in various sectors [62]. Elegbede [49] used xylanases from *Aspergillus niger* and *Trichoderma longibrachiatum* to synthesize silver nanoparticles (Ag NPs). Fungal extracts are rich in bioactive molecules and functional groups, improving the quantitative production of Ag NPs and their stable dispersion [63]. *Pencillium* sp., an endophytic fungus isolated from healthy leaves of *Curcuma longa* (turmeric), used for extracellular production of Ag NPs, showed high antibacterial activity against *Pseudomonas aeruginosa and K. pneumoniae* [64]. The endophytic fungus *Penicillium oxalicum* isolated from *Tecomella undulata* was used to myco-synthesis iron nanoparticles [65].The mechanism that actinomycetes use to transform metallic oxides into metal oxide nanoparticles is uncertain [66]. Compared with the bacterial synthesis of nanoparticles, which requires complex equipment to obtain clear filtrate from the colloidal broth, it is more advantageous. This advantage is further underscored by the exceptional heavy metal tolerance of fungi, their ability for large-scale cultivation, and the production of extracellular nanoparticles that reduce downstream expenses [56].

## 3. Role of Nanoparticles under Abiotic Stress Conditions

Nanomaterials have wide applications in improving agriculture, sustainable crop production, boosting plant metabolism, reducing the loss of nutrients, and alleviating the damage of biotic and abiotic stresses, thereby enhancing yields. Nanoparticles of zinc, copper, iron, selenium, titanium, gold, silver, and selenium have been reported to impact growth and development by influencing morphological, physiological, and biochemical factors in plants under abiotic stresses, as summarized in Table 1. Treatment with nanoparticles reduces the effect of heat stress by increasing hydration ability, chlorophyll content, and stomatal opening regulation. Nanoparticles improve electrolyte leakage, photosynthetic activity, and membrane damage under cold-stress conditions. Wang et al. [67] reported that the foliar application of nanoparticles significantly increased growth parameters and nutrient content while also decreasing malondialdehyde (MDA) and reactive oxygen species (ROS). The accumulation of osmo-protectants, such as soluble carbohydrates, proline, and amino acids, enhanced cold stress tolerance in banana plants. Foliar application of nanoparticles can be a sustainable and economically feasible approach to achieving cold stress tolerance. Nanoparticles alleviate flooding stress by regulating amino acid synthesis, proteins, glycolysis, and ROS mechanisms. Nanoparticles suppress the negative effects of salinity in plants by maintaining chlorophyll content, leaf relative water content, and relative water protection, increasing nitrogen and phosphorus content and uptake, and decreasing sodium content. Nanoparticles are known to enhance the activity of antioxidant enzymes like superoxide dismutase (SOD), catalase (CAT), and peroxidase (POX). An et al. [60] examined the impact of priming seeds with cerium oxide nanoparticles (PNC) on cotton seedling morphological, physiological, biochemical, and transcriptional traits under salinity stress. PNC seed priming significantly increased root length, fresh weight, dry weight, modified root anatomical structure, and vitality. It also decreased reactive oxygen species (ROS) accumulation in seedling roots and alleviated root morphological and physiological changes, suggesting that ROS and conserved Ca^2+^ plant signaling pathways play pivotal roles in PNC-induced salinity tolerance improvement. Plants develop a complex antioxidant system, including enzymatic components like SOD and CAT, which play a crucial role in ROS detoxification under stress. Spraying salt-stressed mango trees with nZnO and nSi significantly increased the activity of SOD, POX, and CAT [68]. Zhang et al. [69] investigated the interaction between cerium oxide nanoparticles (CeO2 NPs) and spinach plants. The foliar spray of CeO_2_ NPs led to a significant decrease in zinc and calcium levels in leaves and roots. Nanoparticles are known to diminish the negative effects of drought stress by increasing the rate of photosynthesis, stomatal conductance, and water uptake efficiency. Nanoparticles are also involved in reducing heavy metal bioavailability and toxicity in plants. Nano-fertilizers increase plant resource use efficiency and help reduce environmental and soil toxicity.

Nanoparticles have also been involved in soil remediation. The closure of industrial and mining facilities has revealed widespread soil contamination globally [70,71,72]. Potentially toxic elements like Pb, As, Cu, or Zn released during industrial processes pose significant threats to human health and ecosystems [73,74,75,76,77]. To address these challenges, nano-remediation is a promising strategy to deal with toxic compounds like pesticides, chlorinated solvents, halogenated chemicals, or heavy metals. Nanoparticles based on iron oxides, phosphates, and goethite have been used to reduce the harmful effects of toxic elements and for environmental remediation, as they are effective in immobilizing and sequestering contaminants like As and Cd through sorption processes [78,79,80]. The application of nanoparticles has been reviewed by Tariq et al. [81] and Al-Khayri et al. [82].

## 4. Mechanisms of Nano-Microbe Interactions

**Figure 1:**
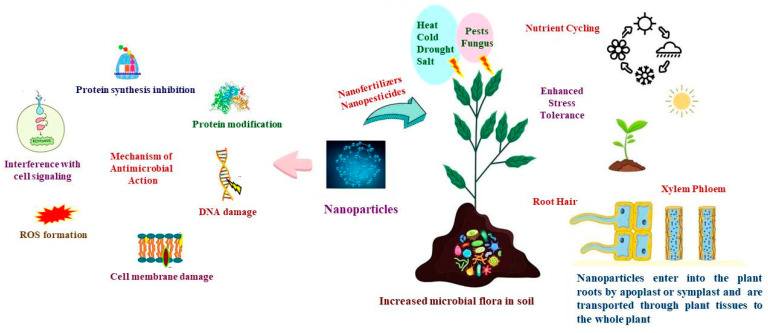
Antimicrobial action of nanoparticles and nano-microbe interaction in soil leads to improved soil productivity and imparts stress tolerance in plants.

Nanoparticles are known to affect plant vigor, microbes, and soil health. Nanoparticles supply essential major and minor elements for the proper growth and development of the plant as well as for the sustainability of soil health. Nanoparticles delivered to the plant through the rhizosphere have to pass through diverse microbial biofilms and varying soil factors that influence nanoparticles dissolution, coating, agglomeration, and bioavailability, as corroborated by [83]. Nanoparticles affect the soil environment, triggering nutrient mineralization and mobilization through numerous integrated mechanisms mediated by plant root exudates, soil organic matter, and rhizospheric bacteria [84]. The interplay between nanoparticles and rhizobacteria can improve soil productivity and plant performance under challenging environmental conditions and assist in developing environmentally safe nano-formulations for agricultural applications (Figure 1).

The cell wall pore size, nanoparticle size, and surface characteristics affect the transportation of nanoparticles [85,86]. The roots may be the major organ of exposure to nanoparticles since they are likely to collect in the soil as a result of drainage, whereas some nanoparticles can be spread directly onto leaves by foliar application. Through their roots, plants have intense interactions with their surroundings, absorbing water, nutrients, and other substances. Since the root’s pore width ranges from 5 to 20 nm, the size of the nanoparticles has the greatest influence on how well they are absorbed via the root [87]. Confocal studies examined the protection of wheat roots from *Pseudomonas chlororaphis* using CuO nanoparticles. Fluorescent Zein Nanoparticles (ZNPs) translocation was observed along the root system’s epidermal layer, cortex, and endodermis. ZNPs revealed that dosage-dependent variations in nanoparticle concentrations adhered to the roots. ZNP translocation across the plant vascular system is hampered due to the larger size of ZNPs [87]. Rodrigues et al. [88] reported that they can be divided into two groups based on the route of nanoparticles exposure to terrestrial plants. Nanoparticles enter the plant system through exposed roots, simulating environmental soil pollution. Moreover, because of their potential advantages in agriculture, nanoparticles have also been sprayed onto crops via foliar spray to promote plant growth and control disease. The method of NP exposure has a considerable impact on the uptake and storage of particles by terrestrial plants. The bioaccumulation factor of Ag and Ce in plants treated with nanoparticles was higher than the corresponding soil values [89]. The surface modification did not impact Ce translocation from roots to shoots [90]. The root Fe content in nanoparticle-treated wheat was three-fold higher than with citric acid-coated nanoparticles [91]. Nanoparticles can move along apoplastic pathways, reaching the cortex. Nhan and his co-workers detected aggregated nanoparticles through symplastic in xylem sap from CeO_2_ NP-treated cotton and CuO NP-treated maize [92]. According to Ji et al. [93], rice treated with TiO_2_ NPs had TiO_2_ NPs on the chloroplast membranes. Additionally, CeO_2_ nanoparticles were discovered in the chloroplast, vacuole, and plasma membrane of cotton grown under hydroponic conditions with various CeO_2_ concentrations. In situ nanoparticles speciation in plants advances our understanding of probable pathways for nanoparticle accumulation and phytotoxicity [94]. Li and co-workers investigated the toxic effects of ZnO nanoparticles on *Deinococcus radiodurans*, a bacterial cell resistant to radiation, and revealed significant internalization, concentration-dependent toxicity, and DNA damage [95]. Singh and co-workers reported downregulation of DNA repair genes and metabolic pathway-related genes [96]. Nanoparticle uptake and action occur exclusively through stomata and extracellular adsorption in leaf air spaces. SiO_2_ NPs have been reported to have the potential to serve as an inexpensive, efficient, safe, and sustainable alternative for plant disease protection [97]. According to an in vitro analysis of copper, silver, and zinc nanoparticles, Cu-NPs were the most successful at preventing the growth of fungi, followed by ZnO NPs. ZnO NPs and Cu NPs were more toxic to fungi than ZnSO_4_ and Cu(OH)_2_, respectively [98].

The characteristics of root exudates, the rhizosphere’s microbiology, and soil health are all expected to be impacted by nano-materials used in practices related to agriculture. A minor shift in the community of microbes may possess an enormous influence on the structural integrity of plants and soil because the condition of plants relies upon the availability of vital micro- and macronutrients, which are fixed, solubilized, or recycled using soil microbes [99]. The majority of soil enzymes are discharged extracellularly from the community of microbes living underneath the rhizospheres of plants, and they aid in the recycling of necessary nutrients that promote microbial development into the soil while enhancing the yield of crops [100]. Numerous chemicals, pesticides, sludge, and fertilizers may be incorporated into the soil, which could significantly impact soil health. The microbiological status of soil may contribute to interruptions in its enzymatic activities [101]. Nevertheless, incorporating nano-compounds in appropriate quantities can boost the soil’s microbiological condition and safeguard soil health [102,103]. The plant growth-promoting rhizobacteria, together with nano zeolite and nano chitosan, increased the activity of the fluorescein diacetate hydrolase enzyme and alkaline phosphatase in Fenugreek [104]. *Zea mays* was examined for the effects of nano silicon dioxide and plant growth-promoting rhizobacteria on plant growth and its rhizospheric microbes. Catalase and peroxidase activities rose substantially more than control when plants were treated with the bacterial consortium and nano silicon dioxide formulation. The enzyme activities such as fluorescein diacetate, dehydrogenase, and alkaline phosphatase were found to be higher in the treated (consortium and nano silicon dioxide combined application) soil [103]. In maize plants, the application of Nanophos, a biofertilizer enriched with phosphate-solubilizing bacteria, not only converts insoluble phosphate into a readily available form, promoting amplified growth and productivity, but also augments the soil’s overall health by bolstering its microbial population [105]. In the nano-formulation of bioinoculants and nanochitosan, soil enzyme activity, including dehydrogenase, fluorescein diacetate, and alkaline phosphatase, was higher. Proteobacteria, Actinobacteria, Chloroflexi, and Firmicutes were the most prevalent bacterial species, indicating a high concentration of rhizobacteria that promotes healthy plant growth [106]. A bioinoculant (*Bacillus* sp.), nano zeolite, and nano chitosan were employed in the maize rhizosphere of the field. There was increased bacterial diversity and FDA (fluorescein diacetate hydrolysis), dehydrogenase, and alkaline phosphatase activity [107]. The activity of alkaline phosphatase, fluorescein diacetate hydrolysis, and dehydrogenase was higher in the combined application of Bacillus spp. and nano zeolite on maize [108]. Fe NPs were reported to impact the bacterial proliferation of *Bacillus aryabhattai*, a plant growth-promoting rhizobacterium [109]. Coinoculation of *Mesorhizobium ciceri* with helper bacteria, *Bacillus subtilis*, and Mo nanoparticles enhanced the symbiotic efficiency of ST282 [110]. It follows that the microorganisms and microbial activity of the rhizosphere are profoundly affected by the application of nano-biofertilizers. It can be concluded that nano-biofertilizers are an intriguing candidate for crop health and growth promotion.

## 5. Nano-Fertilizers

Nano-fertilizer is a substance enclosed in nanoparticles that facilitates the controlled release of nutrients to plants. Nano-fertilizers provide necessary nutrients to plants at the nano-scale with the intent to stimulate plant development while boosting their yield [111,112,113,114,115,116]. The ability of nano-fertilizers to provide plant nutrients with significant absorption rates, increased usage efficiency, and little wastage renders them an excellent choice for facilitating nutrient uptake by plants [1]. According to their needs, nano-fertilizers can be divided into two categories: macronutrients (N, P, and K) and micronutrients (vitamins and Fe, Zn, Ag, Cu, and Au) [117]. Numerous categories of nano-fertilizers have been employed, and various nanoparticles have previously been advocated for use in agriculture. There are several different encapsulation techniques, such as encasing nutrients with a thin protective covering of polymer, coating nutrients with nano-materials like nanotubes, and formulations that can offer nutrients as nano-scale emulsions [118]. There is advancement in the use of urea-coated hydroxyapatite by employing it as a nano-fertilizer [119]. The effectiveness of slow-release fertilizers as a long-term source of nutrients that support crop growth has been demonstrated [120]. The effects of nanoparticles and nano-fertilizers on various crops have been illustrated in Table 2.

### 5.1. Effect of Nano-Fertilizers on Plant Disease

Pest and disease devastation, along with climate change, have an impact on the production of food [141]. Pests and plant pathogens also have a significant negative impact on the production of food. Phyto-pathogens are estimated to reduce yields by 25% in developing nations [142]. Fungi are the most prevalent among them, followed by bacteria, viruses, and nematodes [143]. Nano-materials research is predominantly oriented towards evaluating their effectiveness in disease prevention. Nanoparticles can be used as shields or transporters for delivering active chemicals to defend plants from pathogens like insects, fungi, bacteria, and viruses [144]. Due to their tiny size, positive charge, and vast surface area, nanoparticles have high reactivity and can quickly invade plant cells through the foliage, brand, trunk, root, or persist-on plant components [145,146]. Plant pathogens, including *Phoma destructive*, *P. infestans*, *Rhizopus stolonifer*, *Mucor plumbeus*, *Fusarium oxysporum*, *Botrytis cinerea*, *P. cubensis*, *P. syringae* pv. *lachrymans*, and *Colletotrichum* spp., can be controlled by nanoparticles of Cu, ZnO, MgO, FeO, and Ag. Furthermore, they may regulate the active chemicals that are discharged when employing nanoparticles as carriers, which improves absorption, promotes the availability of water and nutrients, and diminishes their adverse impacts on the environment [147,148]. With their long shelf lives, tailored absorption, dispersion, minimal soil dissolution, and toxic consequences, nano-pesticides effectively prevent plant diseases and promote plant development [149]. The nano-sized silica-silver was made by radioactively irradiating a solution containing silver salt, silicate, and water-soluble polymer to produce nano-silver in conjunction with silica molecules and water-soluble polymer. The nanosized silica-silver depicted varying levels of antifungal activity when used against the investigated fungi that were phyto-pathogenic. In contrast, several helpful bacteria or pathogenic bacteria found in plants were not considerably devastated [150]. *Streptomyces griseus* was used to biosynthesize copper nanoparticles extracellularly. The field efficacy of soil-applied copper nanoparticles was evaluated in *P. hypolateritia*-infected tea plants. Plants administered with nano-copper revealed the highest leaf yield, reduced disease symptoms, and enhanced soil macronutrients [151]. Tomato plants exposed to iron nanoparticles (Fe NPs) showed increased levels of photosynthetic pigments, osmolytes, antioxidant enzymes, POX, polyphenol oxidase (PPO), CAT, and SOD, and the Fe NPs were found to be effective against *Fusarium oxysporum* [152]. The nano-fertilizers (Zn, Fe, and Fe-Zn oxide) were effective in controlling the root knot nematode *Meloidogyne incognita* on tomato plants [153].

### 5.2. Effect of Nano-Fertilizers on Nutrient Enhancement of Crop

The global population is suffering from a deficiency of micronutrients, commonly known as “hidden hunger,” which affects more than 2 billion people [154]. Nano-fertilizers work effectively in delivering nutrients precisely when they are required for the development of plants and can supply nutrients to the crop as a whole [155]. Increased nutrient toxicity may hinder crop growth when using higher fertilizer dosages on crops [117]. The Zn levels in tomato fruits were reportedly elevated in acidic and calcareous soil by applying ZnO-NPs nano-fertilizer [156]. The nutritional value of tomato fruits was considerably increased with the application of a mixed nano-fertilizer [157]. NPK nano-fertilizers are applied at lower rates than the recommended levels (NPK) of chemical fertilizers (conventional fertilizers) and increase crop yield, fertilizer uptake, and dry weight [158]. NAC-urea-Zn can be employed at a lower rate of Zn to promote the delivery of Zn and N [159]. *Ipomoea aquatica* treated with nano-fertilizer revealed greater P and K retention [160]. The use of nano- and chemical phosphatic fertilizers considerably enhanced the nutritional content and uptake of pearl millet. The use of a nano-phosphatic fertilizer resulted in the highest NPK content and absorption, as well as protein content and uptake [161].

### 5.3. Effect of Nano-Fertilizers on Metabolic Activity of Micro-Organisms

The nutrients can be released gradually and continuously by nano-fertilizers, promoting plant growth while safeguarding the variety of microbiota. The phytomicrobiome may be able to successfully minimize biotic and abiotic stress instances with these characteristics [162]. Nanoparticles are recognized as possessing numerous advantages, but their introduction into other habitats poses a possible ecological risk [163]. Nano-fertilizers could possibly have detrimental effects on microbial communities that have been advantageous to the soil and plants [164]. Since the soil, root, and leaf are the primary locations where nano-fertilizers are employed, it is imperative to comprehend the consequences of nano-scale fertilizers on these sites. The prolonged impacts on the environment surrounding plants and, subsequently, the economic viability of nano-fertilizers are decided by the implications of nano-fertilizers on microorganisms dwelling within the ecosystem. The direct application of Ag NPs to soil improved microbial metabolic activity and elevated proteobacterial and acidobacterial density. Increased concentration, however, has a harmful effect on microbial cells [165]. Ag NPs greatly decreased the quantity of bacterial and archaeal amoA genes in soil and significantly altered the organization of the fungal community [166]. Acidobacteria, Actinobacteria, Cyanobacteria, and Nitrospirae considerably diminished with the application of Ag NPs; however, numerous other phyla, such as Proteobacteria and Planctomycetes, increased and predominated [167]. Cota-Ruiz and co-workers examined the growth and development of plants at the physiological and molecular levels after producing lucerne (*Medicago sativa*) in potting soil enriched with large-sized (bulk), nano, and ionic copper compounds. The percentage of all microorganisms (Bacteroidetes, Actinobacteria, Sacharibacteria, and Acidobacteria) participating in elemental acquisition rose substantially in relation to bulk/nano Cu than ionic Cu [168]. Prada and co-workers evaluated the herbicides atrazine (ATZ) and copper nanoparticle (NCu) administered sequentially, which influenced the diversity of all bacteria as well as the nitrifying populations of ammonia-oxidizing archaea and ammonia-oxidizing bacteria [169]. In order to assess their effects on soil enzyme activities (invertase, urease, catalase, and phosphatase) and bacterial communities in black soil and saline-alkali soil, four metal oxide nanoparticles—magnetite (nFe_3_O_4_), titanium dioxide (nTiO_2_), cerium dioxide (nCeO_2_), and zinc oxide (nZnO)—were used. nZnO exhibited a greater impact on the enzymatic activities of soil over nTiO_2_, nCeO_2_, and nFe_3_O_4_, and saline-alkali soil proved more vulnerable to metal oxide nanoparticles than black soil. The pyrosequencing studies of samples of saline-alkali soil treated with nZnO showed substantial variability in the diversity among their bacterial community, including Bacilli, Alphaproteobacteria, and Gammaproteobacteria [170]. In some investigations, the number of soil microorganisms treated with nano-fertilizer became substantially elevated, whereas, in some cases, higher dosages of nano-fertilizer interfered with the symbiosis among plants and bacteria, leading to a detrimental effect on plant development. Environmental risks and repercussions are complicated to measure and are insufficiently comprehended [171].

## 6. Nano-Biofertilizers

**Figure 2:**
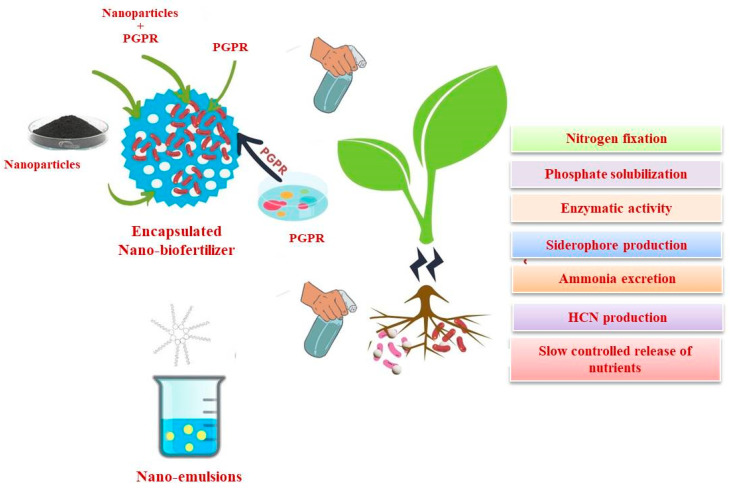
Nano-biofertilizer formed by encapsulating biofertilizers into nanomaterial which are then applied to plants for improving growth and productivity.

Nanoparticles and biofertilizers are incorporated to produce a nano-biofertilizer by encapsulating biofertilizers inside a compatible nanomaterial. In addition to eliminating the negative impacts of environmental challenges, they manage the penetration of essential nutrients through the soil. They boost accessibility and intake of vitamins and minerals, utilize less chemical fertilizer, are cost-efficient, and are environmentally benign [172,173]. A nano-composite biofertilizer (NCB) made of acylated homoserine lactone (AHL)-coated Fe-carbon nano-fibers (Fe-CNFs) and bacterial (*Panebacillus polymyxa*) endospores imprisoned in activated carbon beads has been developed and applied to leguminous (*Cicer arietinum*) and non-leguminous (*Triticum aestivum*) plants, which showed elevation in protein content, biomass, root length, and chlorophyll content of the plants. The plants produced utilizing NCB further showed competence to endure wheat root rot imposed by *Fusarium oxysporum* f. sp. *Cicero* and *Fusarium* wilt of chickpea prompted by *Cochliobolus sativus*, respectively [174]. Nano silver and nitroxin biofertilizers were reported to boost potato tuber output [175]. The study on the effects of nano-biofertilizers composed of (Biozar^®^) containing *Azotobacter* and *Pseudomonas* bacteria and nano-fertilizers such as Fe, Zn, and Mn revealed an improvement in the length, quantity, and weight of spikes, the number of seeds in each spike, and the days prior to physiological maturity in early-planting wheat [176]. Nano-biofertilizer reportedly increased the maize grain yield [177]. The impact of nano (iron chelate nano-fertilizer, potassium chelate nano-fertilizer, and combination of iron and potassium chelate fertilizers) and biofertilizers (Azetobarvar, Phosphatebarvar, and combination of Azetobarvar and Phosphatebarvar) was investigated in relation to the forage sorghum. The combined application of biofertilizers (azetobarvar + phosphorbarvar) + chelated nano-fertilizers (Fe and K) treatments produced high levels of chlorophyll a, chlorophyll b, carotenoids, and carbohydrates [178]. Chitosan-immobilized silica nano-composites were found to boost tomato production, extend tomato fruit longevity, and conserve water [179]. Ag-nanoparticles and plant growth-promoting rhizobacteria treatment collectively increased the total quantity of flavonoids in leaves and the activities of phenylalanine ammonia-lyase, superoxide dismutase, and catalase over control in plant leaves [180]. The protein content of the bulb increased substantially when Ag NPs and PGPR were used, whereas the leaf flavonoids were reduced and the bulb flavonoid content was considerably elevated [181]. Plants may benefit from the slow-release properties of nano-biofertilizer, improved functional component stability, use of modest doses, minimal nutrient loss through leaching and degradation, concealing soil nutrient exhaustion, and improved crop production qualities. The impact of nano-fertilizers on plants is depicted in Figure 2.

Nano-biofertilizers have been developed with cutting-edge approaches that can be considered beneficial to the environment and primarily rely on biological components rather than hazardous chemicals. Due to their ability to generate inorganic materials intracellularly or extracellularly, microbes play a crucial role in the production of such nanoparticles, employing diatoms for siliceous materials, magnetostatic bacteria for magnetite, and S-layer bacteria for gypsum and calcium carbonate [182]. In the formulation of nano-biofertilizer, biofertilizer is impregnated with nano-scale polymers. In addition to essential nutrients for plant growth, the biofertilizer also includes bacteria that aid plant growth. This approach provides essential nutrients for plant growth and incorporates beneficial bacteria that aid in plant development. By utilizing nano-scale polymers, the study ensures a controlled and sustained release of nutrients and microbes to enhance plant life [183]. The biofertilizer aspects comprised of rhizobium, which encourage plant growth, are shielded by nano-encapsulation technology, which is also used to prolong the longevity of the biofertilizer components and facilitate gradual liberation of the rhizobium [184]. In order to create nano-biofertilizers, rhizobacteria that promote plant development and associated metabolites were nano-encapsulated employing carbon nanotubes along with alginate-silica nanoparticles [185]. To encapsulate *Bacillus velezensis*, sodium alginate-gelatin microcapsules rich in carbon nanotubes and SiO_2_ nanoparticles were developed [186]. The encapsulation process prolongs the longevity of the biofertilizer components and allows for a gradual release of rhizobium. This ensures that the beneficial microbes are effectively delivered to the plant roots, enhancing their overall growth and health. A formulation using alginate-Arabic gum and SiO_2_/TiO_2_ nanoparticles enhances the bio-control activity of *Streptomyces fulvissimus* [187]. Encapsulation boosts colonization and provides protection in harsh environments. This approach ensures prolonged effectiveness in adverse conditions. The controlled release promotes increased agent colonization in soil, ultimately improving microbial biocontrol activity and soil health. Salicylic acid and zinc oxide nanoparticle-augmented alginate beads were used to entrap *Pseudomonas* sp. DN18, and this formulation is reported to have potential for implementation in agriculture [188]. The interaction between the nanoparticles and the microbial agent enhances the stability and efficacy of the bio-control agent. A titanium nanoparticle-enriched alginate-bentonite film containing *Bacillus subtilis* Vru1 has been developed [189]. The incorporation of nanoparticles enhances the properties of the film, which in turn affects the interaction between the microbial agent and the soil environment. This formulation has the potential to improve soil health by introducing beneficial microorganisms with enhanced stability and protection. For the development of two-layer and multilayer assemblages that function as innovative, intelligent micro-encapsulation frameworks, *Bacillus velezensis*, enclosed in alginate mixed using whey protein, zedo, mastic, and tragacanth gums, along with silica and titania, was able to generate auxin, lipase, siderophore, protease, and disintegration of mineral phosphate. It can be used to develop a suitable bio-fertilizer formulation based on naturally decomposing compounds as an environmentally friendly and cost-effective substitute for chemical fertilizers [190]. The microcapsules can be used to load biological agents against biotic stressors and protect microorganisms from environmental influences. These formulations enhance the stability, protection, and efficacy of microbial agents, leading to improved bio-control activity, nutrient release, and overall soil health. This approach represents a promising development in biological plant disease management and sustainable nutrient delivery. Thus, in the future, the application of these formulation approaches may represent a new development in the biological management of plant diseases and in the slow and site-specific release of nutrients. Nanoparticle-based farming holds promise as an economical and environmentally benign strategy for the growth of sustainable agribusiness [104].

## 7. Conclusions and Future Prospects

Nanotechnology is revolutionizing agricultural technology by unlocking new frontiers of innovation. It allows for enhanced nutrient delivery, precise pest and disease management, improved soil health, and optimized resource utilization. The intricate manipulation of matter at the nano-scale level offers a toolkit of novel solutions, paving the way for sustainable and resilient food production systems in the face of evolving challenges and global demands. It might boost agricultural productivity by establishing an improved complementary interaction between the consumption of chemicals and stability in the environment. In the near future, it might be used as cutting-edge science to address the dilemma of worldwide malnutrition. Sophisticated nano-technological tools for innovative agriculture could result in increased output with lower expenditure on goods and services. In order to treat plant diseases and reduce crop loss, nanotechnology has made it more convenient to produce modern, effective agricultural products for plants, including nano-fertilizers, nano-biofertilizers, and nano-pesticides. Even though there is a plethora of literature on particular nanoparticles, the extent of the toxicity of numerous nanoparticles remains uncertain. As a result, the potential uses of such substances are restricted because of the dearth of comprehension of risk evaluations and impacts like toxicity, bioaccumulation, water and soil contamination, biodiversity impact, and disruption of ecosystem functions. Unintended consequences and regulatory challenges add to the concerns about the well-being of humans and other life forms. International regulatory and legal collaborations are required to fully utilize this modern technology, diligent database, and monitoring design.

Nanotechnology in agriculture, particularly nano-fertilizers and nano-biofertilizers, offers a promising future. Advanced delivery systems for fertilizers and nutrients, such as nano-carriers and nano-capsules, can be explored. The involvement of nanoparticles in precision agriculture techniques, including IoT sensors (the Internet of Things) and data analytics, holds immense potential. The sustainability and environmental impact of nanoparticles with long-term effects on soil health, water quality, and biodiversity need to be assessed. The development of novel targeted soil and crop-specific hybrid nano-formulations as fertilizers and pesticides with cost-benefit analysis requires consideration. Regulation and commercialization of nanotechnology-based agri-products have to be investigated with case studies and success stories. Interdisciplinary approaches and global perspectives will be highlighted and public awareness and education will be crucial in promoting adoption of nanotechnology.

## Figures and Tables

**Table 1:** Morphological, physiological, and biochemical effects imparting abiotic stress tolerance in plants in response to application of nanoparticles.

Morphological Effects	Physiological Responses	Biochemical Responses
Increased plant height Improved plant growth, root and shoot length Improved leaf area, leaf number, and leaf development Increased dry weight, fresh weight, and biomass production Enhanced seedling weight Improved photosynthesis Increased seed yield Improved fruit/grain quality and size	Reduced MDA production and H_2_O_2_ accumulation Reduced electrolyte leakage Reduced lipid peroxidation Reduced chlorophyll damage Enhanced photosynthetic pigments Improved photosynthesis Improved nitrogen metabolism Enhanced nutrient uptake Enhanced mineral uptake Reduced heavy metal uptake and accumulation	Reduced ROS and MDA production Improved chlorophyll and phenol contents Regulated accumulation of proline, glutathione, and phytochelation Improved seed protein/fiber content and seed oil

**Table 2:** Effects of nanoparticles and nano-fertilizers and their effective doses on various crops.

Nanoparticles/ Nano-Fertilizers	Crop	Active Dose	Effects	References
Zinc NPs	Pearl millet	10 mgL^−1^	Increased shoot length, root length, root area, chlorophyll content, total soluble leaf protein, plant dry biomass, grain yield, and enzyme activities.	[121]
Nano chitosan-NPK	Wheat	10%	Boosted harvest index, crop index, mobilization index; shorter lifespan with nano-fertilization; and improved plant development and yield.	[122]
ZnO NPs	Mung bean	10 mgL^−1^	Improved P-solubilizing enzyme activity, phosphorus intake, stem height, root length, chlorophyll and protein levels, and enhanced microbial communities in the rhizosphere.	[123]
ZnO NPs	Cotton	200 mgL^−1^	Increased growth, total biomass, carotenoids, chlorophyll a, chlorophyll b, total soluble protein, reduced MDA content, enhanced enzyme activities (SOD, POX, CAT).	[124]
Fe_2_O_3_ NPs	Peanut	2–1000 mgKg^−1^	Enhanced root length, plant height, biomass, SPAD values, increased Fe content, controlled phytohormones, and antioxidant enzymes.	[125]
Ag NPs	Fenugreek	40 mgL^−1^	Improved growth parameters, biochemical properties (pigments, IAA), increased pod and seed production, higher contents of phenolics, flavonoids, tannins, and antioxidant activity.	[126]
TiO_2_, Fe_2_O_3_, CuO NPs	Wheat	50 and 500 mgKg^−1^	CuO NPs reduced growth, Fe_2_O_3_ NPs improved precocity, TiO_2_ NPs enhanced amino acid content and nutritional value, increased Cu contents, increased Fe content, and decreased Zn content in grains. TiO_2_ and Fe_2_O_3_ improved amino acid content.	[127]
Ionic gelled NPKS NPs	Maize	40 ppm	Enhanced growth: plant height, leaves, and chlorophyll content	[128]
Ag NPs	Onion	2000 ppm	Improved morphological, yield, and quality aspects.	[129]
Fe_3_O_4_ NPs	Tomato	50 mgKg^−1^	Improved iron content in shoot, enhanced plant growth and yield, better growth, and elevated iron content.	[130]
ZnO-MnO-NPs, FeO-ZnO-NPs	Andean lupin, cabbage	270 ppm	Improved height, root size, chlorophyll content, and biomass.	[131]
Feo NPs	Lettuce	1–5 ppm	Increased seedling root elongation.	[132]
γ-Fe_2_O_3_	Maize	20 mgL^−1^	Enhanced germination index and vigor index. Increased root elongation.	[133]
ZnONPs	Maize	1500 ppm	Increase germination percentage and vigor index.	[134]
MnNPs	Mung bean	0.05 mg/L	Improved root and shoot lengths.	[135]
Nano selenium	Tomato	100 mgL^−1^	Activation of antioxidant enzymes (CAT, POX), enhanced yield and quality of fruits, and increased soluble solids content.	[136]
Nano urea	Mung bean	500–1000 mgL^−1^	Enhanced protein content, free radical scavenging activity, and phenolic content reduced in nitrate leaching, increased plant growth attributes, and crop biomass.	[137]
Nano gypsum	Spinach	240 kg/ha	Enhanced growth in saline-sodic soil.	[138]
Nano phosphorus	Fenugreek	0.1 gL^−1^	Increased drought stress tolerance, enhanced plant growth and productivity, increased water use efficiency, osmo-regulatory compounds, soluble sugars and proline, and activation of antioxidant enzymes.	[139]
Nano copper	Wheat	100 mgL^−1^	Amelioration of DNA damage and methylation.	[140]

## Data Availability

Not applicable.

## References

[1] Fatima F., Hashim A., Anees S. (2021). Efficacy of nanoparticles as nanofertilizer production: A review. Environ. Sci. Pollut. Res..

[2] Iqbal M.A. (2019). Nano-fertilizers for sustainable crop production under changing climate: A global perspective. Sustain. Crop Prod..

[3] Speight J.G. (2017). Sources and types of organic Pollutants. Environmental Organic Chemistry for Engineers.

[4] Ostadi A., Javanmard A., Machiani M.A., Morshedloo M.R., Nouraein M., Rasouli F., Maggi F. (2020). Effect of different fertilizer sources and harvesting time on the growth characteristics, nutrient uptakes, essential oil productivity and composition of *Mentha* x *piperita* L.. Ind. Crops Prod..

[5] Chatzistathis T., Fanourakis D., Aliniaeifard S., Kotsiras A., Delis C., Tsaniklidis G. (2021). Leaf age-dependent effects of boron toxicity in two *Cucumis melo* varieties. Agronomy.

[6] Xu T., Wang Y., Aytac Z., Zuverza-Mena N., Zhao Z., Hu X., Demokritou P. (2022). Enhancing agrichemical delivery and plant development with biopolymer-based stimuli responsive core–shell nanostructures. ACS Nano.

[7] Akhtar N., Ilyas N., Meraj T.A., Pour-Aboughadareh A., Sayyed R.Z., Mashwani Z.U.R., Poczai P. (2022). Improvement of plant responses by nanobiofertilizers: A step towards sustainable agriculture. Nanomaterials.

[8] Rameshaiah G., Pallavi J., Shabnam S. (2015). Nano fertilizers and nano sensors—An attempt for developing smart agriculture. Int. J. Eng. Res. Gen. Sci..

[9] Dimkpa C.O., Bindraban P.S. (2017). Nanofertilizers: New products for the industry?. J. Agric. Food Chem..

[10] Duhan J.S., Kumar R., Kumar N., Kaur P., Nehra K., Duhan S. (2017). Nanotechnology: The new perspective in precision agriculture. Biotechnol. Rep..

[11] Ali M.A., Rehman I., Iqbal A., Din S., Rao A.Q., Latif A., Husnain T. (2014). Nanotechnology, a new frontier in Agriculture. Adv. Life Sci..

[12] Wang Y., Borgatta J., White J.C. (2022). Protecting foods with biopolymer fibres. Nat. Food.

[13] Wang Y., Deng C., Rawat S., Cota-Ruiz K., Medina-Velo I., Gardea-Torresdey J.L. (2021). Evaluation of the effects of nanomaterials on rice (*Oryza sativa* L.) responses: Underlining the benefits of nanotechnology for agricultural applications. ACS Agric. Sci. Technol..

[14] Ghidan A.Y., Al Antary T.M. (2019). Applications of Nanotechnology in Agriculture.

[15] Wang Y., Deng C., Elmer W.H., Dimkpa C.O., Sharma S., Navarro G., Wang Z., LaReau J., Steven B.T., Wang Z. (2022). Therapeutic delivery of nanoscale sulfur to suppress disease in tomatoes: In vitro imaging and orthogonal mechanistic investigation. ACS Nano.

[16] Tan W., Deng C., Wang Y., Peralta-Videa J.R., Gardea-Torresdey J.L. (2019). Interaction of Nanomaterials in Secondary Metabolites Accumulation, Photosynthesis, and Nitrogen Fixation in Plant Systems, in Comprehensive Analytical Chemistry.

[17] Khan I., Saeed K., Khan I. (2019). Nanoparticles: Properties, applications and toxicities. Arab. J. Chem..

[18] Pilarska A., Wysokowski M., Markiewicz E., Jesionowski T. (2013). Synthesis of magnesium hydroxide and its calcinates by a precipitation method with the use of magnesium sulfate and poly (*Ethylene glycols*). Powder Technol..

[19] Awad M., Yosri M., Abdel-Aziz M.M., Younis A.M., Sidkey N.M. (2021). Assessment of the antibacterial potential of biosynthesized silver nanoparticles combined with vancomycin against methicillin-resistant *Staphylococcus aureus*—Induced infection in rats. Biol. Trace Elem. Res..

[20] Schröfel A., Kratošová G., Šafařík I., Šafaříková M., Raška I., Shor L.M. (2014). Applications of biosynthesized metallic nanoparticles—A review. Acta Biomater..

[21] Malik P., Shankar R., Malik V., Sharma N., Mukherjee T.K. (2014). Green chemistry based benign routes for nanoparticle synthesis. J. Nanopart..

[22] Moustafa Y., Morsi R.E., Sidkey N.M., Arafa R.A., Elhateir M.M. (2015). Extracellular biosynthesis of Zn (II) nanoparticles by Zn-tolerant *Fusarium nygamai*, F3 Zn/S with antimicrobial activity. Afr. J. Mycol. Biotech.

[23] Wu H., Li Z. (2022). Recent advances in nano-enabled agriculture for improving plant performance. Crop J..

[24] Raliya R., Saharan V., Dimkpa C., Biswas P. (2017). Nanofertilizer for precision and sustainable agriculture: Current state and future perspectives. J. Agric. Food Chem..

[25] Deng C., Wang Y., Cota-Ruiz K., Reyes A., Sun Y., Peralta-Videa J., Hernandez-Viezcas J.A., Turley R.S., Niu G., Li C. (2020). Bok choy (*Brassica rapa*) grown in copper oxide nanoparticles-amended soils exhibits toxicity in a phenotype-dependent manner: Translocation, biodistribution and nutritional disturbance. J. Hazard. Mater..

[26] Wang Y., Deng C., Cota-Ruiz K., Tan W., Reyes A., Peralta- Videa J.R., Gardea-Torresday J.L. (2021). Effects of different surface-coated nTiO_2_ on full-grown carrot plants: Impacts on root splitting, essential elements, and Ti uptake. J. Hazard. Mater..

[27] Thirugnanasambandan T. (2018). Advances and Trends in Nano-Biofertilizers. https://papers.ssrn.com/sol3/papers.cfm?abstract_id=3306998.

[28] Hu X., Li D., Gao Y., Mu L., Zhou Q. (2016). Knowledge gaps between nanotoxicological research and nanomaterial safety. Environ. Int..

[29] Sharma S., Rana V.S., Pawar R., Lakra J., Racchapannavar V. (2021). Nanofertilizers for sustainable fruit production: A review. Environ. Chem. Lett..

[30] Kumari R., Suman K., Karmakar S., Lakra S.G., Saurav G.K., Mahto B.K. (2023). Regulation and safety measures for nanotechnology-based agri-products. Front. Genome Ed..

[31] Tsekhmistrenko S.I., Bityutskyy V.S., Tsekhmistrenko O.S., Horalskyi L.P., Tymoshok N.O., Spivak M.Y. (2020). Bacterial synthesis of nanoparticles: A green approach. Biosyst. Divers..

[32] Das M., Chatterjee S. (2019). Green Synthesis of Metal/Metal Oxide Nanoparticles toward Biomedical Applications: Boon or Bane. Green Synthesis, Characterization and Applications of Nanoparticles.

[33] Sanjay S.S. (2019). Safe Nano is Green Nano. Green Synthesis, Characterization and Applications of Nanoparticles.

[34] Wang L., Ali J., Zhang C., Mailhot G., Pan G. (2020). Simultaneously enhanced photocatalytic and antibacterial activities of TiO_2_/Ag composite nanofibers for wastewater purification. J. Environ. Chem. Eng..

[35] Shah J.H., Fiaz M., Athar M., Ali J., Rubab M., Mehmood R., Jamil S.U.U., Djellabi R. (2019). Facile synthesis of N/B-double-doped Mn_2_O_3_ and WO_3_ nanoparticles for dye degradation under visible light. Environ. Technol..

[36] Manoj D., Saravanan R., Santhanalakshmi J., Agarwal S., Gupta V.K., Boukherroub R. (2018). Towards green synthesis of monodisperse Cu nanoparticles: An efficient and high sensitive electrochemical nitrite sensor. Sens. Actuators B Chem..

[37] Vetchinkina E., Loshchinina E., Kupryashina M., Burov A., Pylaev T., Nikitina V. (2018). Green synthesis of nanoparticles with extracellular and intracellular extracts of basidiomycetes. PeerJ.

[38] Pal G., Rai P., Pandey A. (2019). Green Synthesis of Nanoparticles: A Greener Approach for a Cleaner Future. Green Synthesis, Characterization and Applications of Nanoparticles.

[39] Alprol A.E., Mansour A.T., Abdelwahab A.M., Ashour M. (2023). Advances in Green Synthesis of Metal Oxide Nanoparticles by Marine Algae for Wastewater Treatment by Adsorption and Photocatalysis Techniques. Catalysts.

[40] Nanda M., Kumar V., Sharma D. (2019). Multimetal tolerance mechanisms in bacteria: The resistance strategies acquired by bacteria that can be exploited to ‘clean-up’heavy metal contaminants from water. Aquat. Toxicol..

[41] Godoy-Gallardo M., Eckhard U., Delgado L.M., de Roo Puente Y.J., Hoyos-Nogués M., Gil F.J., Perez R.A. (2021). Antibacterial approaches in tissue engineering using metal ions and nanoparticles: From mechanisms to applications. Bioact. Mater..

[42] Chokriwal A., Sharma M.M., Singh A. (2014). Biological synthesis of nanoparticles using bacteria and their applications. Am. J. PharmTech Res..

[43] Garole D.J., Choudhary B.C., Paul D., Borse A.U. (2018). Sorption and recovery of platinum from simulated spent catalyst solution and refinery wastewater using chemically modified biomass as a novel sorbent. Environ. Sci. Pollut. Res..

[44] Fang X., Wang Y., Wang Z., Jiang Z., Dong M. (2019). Microorganism assisted synthesized nanoparticles for catalytic applications. Energies.

[45] Rautela A., Rani J. (2019). Green synthesis of silver nanoparticles from Tectona grandis seeds extract: Characterization and mechanism of antimicrobial action on different microorganisms. J. Anal. Sci. Technol..

[46] Wang Z., Li Q., Chen Y., Cui B., Li Y., Besenbacher F., Dong M. (2018). The ambipolar transport behavior of WSe_2_ transistors and its analogue circuits. NPG Asia Mater..

[47] Sunkar S., Nachiyar C.V. (2012). Biogenesis of antibacterial silver nanoparticles using the endophytic bacterium *Bacillus cereus* isolated from *Garcinia xanthochymus*. Asian Pac. J. Trop. Biomed..

[48] Iravani S. (2014). Bacteria in nanoparticle synthesis: Current status and future prospects. Int. Sch. Res. Not..

[49] Singh A., Gautam P.K., Verma A., Singh V., Shivapriya P.M., Shivalkar S., Sahoo A.K., Samanta S.K. (2020). Green synthesis of metallic nanoparticles as effective alternatives to treat antibiotics resistant bacterial infections: A review. Biotechnol. Rep..

[50] Alam H., Khatoon N., Khan M.A., Husain S.A., Saravanan M., Sardar M. (2020). Synthesis of selenium nanoparticles using probiotic bacteria *Lactobacillus acidophilus* and their enhanced antimicrobial activity against resistant bacteria. J. Clust. Sci..

[51] Dong Z.-Y., Narsing Rao M.P., Xiao M., Wang H.-F., Hozzein W.N., Chen W., Li W.-J. (2017). Antibacterial activity of silver nanoparticles against *Staphylococcus warneri* synthesized using endophytic bacteria by photo-irradiation. Front. Microbiol..

[52] Rajora N., Kaushik S., Jyoti A., Kothari S.L. (2016). Rapid synthesis of silver nanoparticles by *Pseudomonas stutzeri* isolated from textile soil under optimised conditions and evaluation of their antimicrobial and cytotoxicity properties. IET Nanobiotechnol..

[53] Ramadan M.M., Almoammar H., Abd-Elsalam K.A. (2019). Microbially Synthesized Biomagnetic Nanomaterials. Magnetic Nanostructures. Environmental and Agricultural Applications.

[54] Sharma R., Khokhar M., Jat R., Khandelwal S. (2012). Role of algae and cyanobacteria in sustainable agriculture system. Wudpecker J. Agric. Res..

[55] Kannan R., Stirk W., Van Staden J. (2013). Synthesis of silver nanoparticles using the seaweed *Codium capitatum* PC Silva (*Chlorophyceae*). S. Afr. J. Bot..

[56] Gade A., Ingle A., Whiteley C., Rai M. (2010). Mycogenic metal nanoparticles: Progress and applications. Biotechnol. Lett..

[57] Michael A., Singh A., Roy A., Islam M.R. (2022). Fungal-and algal-derived synthesis of various nanoparticles and their applications. Bioinorg. Chem. Appl..

[58] Tyagi P.K. (2016). Production of metal nanoparticles from biological resources. Int. J. Curr. Microbiol. Appl. Sci..

[59] Sidkey N.M., Arafa R.A., Moustafa Y.M., Morsi R.E., Elhateir M.M. (2017). Biosynthesis of Mg and Mn intracellular nanoparticles via extremo-Metallotolerant *Pseudomonas stutzeri*, B4 Mg/W and *Fusarium nygamai*, F4 Mn/S. J. Microbiol. Biotechnol. Food Sci..

[60] An J., Hu P., Li F., Wu H., Shen Y., White J.C., Tian X., Li Z., Giraldo J.P. (2020). Emerging investigator series: Molecular mechanisms of plant salinity stress tolerance improvement by seed priming with cerium oxide nanoparticles. Environ. Sci. Nano.

[61] Alghuthaymi M.A., Almoammar H., Rai M., Said-Galiev E., Abd-Elsalam K.A. (2015). Myconanoparticles: Synthesis and their role in phytopathogens management. Biotechnol. Biotechnol. Equip..

[62] Gahlawat G., Choudhury A.R. (2019). A review on the biosynthesis of metal and metal salt nanoparticles by microbes. RSC Adv..

[63] Win T.T., Khan S., Fu P. (2020). Fungus-(*Alternaria* sp.) mediated silver nanoparticles synthesis, characterization, and screening of antifungal activity against some phytopathogens. J. Nanotechnol..

[64] Singh D., Rathod V., Ninganagouda S., Herimath J., Kulkarni P. (2013). Biosynthesis of silver nanoparticle by endophytic fungi Pencillium sp. isolated from *Curcuma longa* (*turmeric*) and its antibacterial activity against pathogenic gram negative bacteria. J. Pharm. Res..

[65] Mathur P., Saini S., Paul E., Sharma C., Mehtani P. (2021). Endophytic fungi mediated synthesis of iron nanoparticles: Characterization and application in methylene blue decolorization. Curr. Res. Green Sustain. Chem..

[66] Rajivgandhi G., Gnanamangai B.M., Prabha T.H., Poornima S., Maruthupandy M., Alharbi N.S., Kadaikunnan S., Li W.-J. (2022). Biosynthesized zinc oxide nanoparticles (ZnO NPs) using actinomycetes enhance the anti-bacterial efficacy against *K. pneumoniae*. J. King Saud Univ. Sci..

[67] Wang A., Li J., Al-Huqail A.A., Al-Harbi M.S., Ali E.F., Wang J., Ding Z., Rekaby S.A., Ghoneim A.M., Eissa M.A. (2021). Mechanisms of chitosan nanoparticles in the regulation of cold stress resistance in banana plants. Nanomaterials.

[68] Elsheery N.I., Helaly M.N., El-Hoseiny H.M., Alam-Eldein S.M. (2020). Zinc oxide and silicone nanoparticles to improve the resistance mechanism and annual productivity of salt-stressed mango trees. Agronomy.

[69] Zhang H., Lu L., Zhao X., Zhao S., Gu X., Du W., Wei H., Ji R., Zhao L. (2019). Metabolomics reveals the “invisible” responses of spinach plants exposed to CeO_2_ nanoparticles. Environ. Sci. Technol..

[70] Adriano D.C., Adriano D.C. (2001). Bioavailability of trace metals. Trace Elements in Terrestrial Environments: Biogeochemistry, Bioavailability and Risks of Metals.

[71] Gallego J., Rodríguez-Valdés E., Esquinas N., Fernández-Braña A., Afif E. (2016). Insights into a 20-ha multi-contaminated brownfield megasite: An environmental forensics approach. Sci. Total Environ..

[72] Santucci L., Carol E., Tanjal C. (2018). Industrial waste as a source of surface and groundwater pollution for more than half a century in a sector of the Río de la Plata coastal plain (Argentina). Chemosphere.

[73] Lado L.R., Hengl T., Reuter H.I. (2008). Heavy metals in European soils: A geostatistical analysis of the FOREGS Geochemical database. Geoderma.

[74] Magiera T., Zawadzki J., Szuszkiewicz M., Fabijańczyk P., Steinnes E., Fabian K., Miszczak E. (2018). Impact of an iron mine and a nickel smelter at the Norwegian/Russian border close to the Barents Sea on surface soil magnetic susceptibility and content of potentially toxic elements. Chemosphere.

[75] Fraga C.G. (2005). Relevance, essentiality and toxicity of trace elements in human health. Mol. Asp. Med..

[76] Gopalakrishnan A., Krishnan R., Thangavel S., Venugopal G., Kim S.-J. (2015). Removal of heavy metal ions from pharma-effluents using graphene-oxide nanosorbents and study of their adsorption kinetics. J. Ind. Eng. Chem..

[77] Irem S., Islam E., Maathuis F.J., Niazi N.K., Li T. (2019). Assessment of potential dietary toxicity and arsenic accumulation in two contrasting rice genotypes: Effect of soil amendments. Chemosphere.

[78] Giménez J., Martínez M., de Pablo J., Rovira M., Duro L. (2007). Arsenic sorption onto natural hematite, magnetite, and goethite. J. Hazard. Mater..

[79] Waychunas G.A., Kim C.S., Banfield J.F. (2005). Nanoparticulate iron oxide minerals in soils and sediments: Unique properties and contaminant scavenging mechanisms. J. Nanoparticle Res..

[80] Dong S., Li J., Zhao M. (2010). Influence of phosphate application on rice absorbing and accumulation of Cd in Cd polluted paddy soil. J. Northeast Agric Univ..

[81] Tariq S.L., Ali H.M., Akram M.A., Janjua M.M., Ahmadlouydarab M. (2020). Nanoparticles enhanced phase change materials (NePCMs)—A recent review. Appl. Therm. Eng..

[82] Al-Khayri J.M., Rashmi R., Surya Ulhas R., Sudheer W.N., Banadka A., Nagella P., Aldaej M.I., Rezk A.A.-S., Shehata W.F., Almaghasla M.I. (2023). The role of nanoparticles in response of plants to abiotic stress at physiological, biochemical, and molecular levels. Plants.

[83] Anderson A.J., Britt D.W., Dimkpa C.O. (2023). Nano–microbe interaction and implications for soil health and plant vigor: Dialogs in the rhizosphere. Nano-Enabled Sustainable and Precision Agriculture.

[84] Rajput V.D., Kumari A., Upadhyay S.K., Minkina T., Mandzhieva S., Ranjan A., Sushkova S., Burachevskaya M., Rajput P., Konstantinova E. (2023). Can nanomaterials improve the soil microbiome and crop productivity?. Agriculture.

[85] Du B., Yu M., Zheng J. (2018). Transport and interactions of nanoparticles in the kidneys. Nat. Rev. Mater..

[86] Wang Y., Deng C., Cota-Ruiz K., Peralta-Videa J.R., Sun Y., Rawat S., Tan W., Reyes A., Hernandez-Viezcas J.A., Niu G. (2020). Improvement of nutrient elements and allicin content in green onion (*Allium fistulosum*) plants exposed to CuO nanoparticles. Sci. Total Environ..

[87] Prasad A., Astete C.E., Bodoki A.E., Windham M., Bodoki E., Sabliov C.M. (2017). Zein nanoparticles uptake and translocation in hydroponically grown sugar cane plants. J. Agric. Food Chem..

[88] Rodrigues S., Trindade T., Duarte A., Pereira E., Koopmans G., Römkens P. (2016). A framework to measure the availability of engineered nanoparticles in soils: Trends in soil tests and analytical tools. Trends Anal. Chem..

[89] Ma C., White J.C., Zhao J., Zhao Q., Xing B. (2018). Uptake of engineered nanoparticles by food crops: Characterization, mechanisms, and implications. Annu. Rev. Food Sci. Technol..

[90] Barrios A.C. (2016). Effects of Citric Acid Coated and Uncoated Cerium Oxide Nanoparticles in Tomato (*Solanum lycopersicum*) Plants. Ph.D. Thesis.

[91] Iannone M.F., Groppa M.D., de Sousa M.E., van Raap M.B.F., Benavides M.P. (2016). Impact of magnetite iron oxide nanoparticles on wheat (*Triticum aestivum* L.) development: Evaluation of oxidative damage. Environ. Exp. Bot..

[92] Nhan L.V., Ma C., Rui Y., Liu S., Li X., Xing B., Liu L. (2015). Phytotoxic mechanism of nanoparticles: Destruction of chloroplasts and vascular bundles and alteration of nutrient absorption. Sci. Rep..

[93] Ji Y., Zhou Y., Ma C., Feng Y., Hao Y., Rui Y., Wu W., Gui X., Han Y., Wang Y. (2017). Jointed toxicity of TiO_2_ NPs and Cd to rice seedlings: NPs alleviated Cd toxicity and Cd promoted NPs uptake. Plant Physiol. Biochem..

[94] Castillo-Michel H.A., Larue C., Del Real A.E.P., Cotte M., Sarret G. (2017). Practical review on the use of synchrotron based micro-and nano-X-ray fluorescence mapping and X-ray absorption spectroscopy to investigate the interactions between plants and engineered nanomaterials. Plant Physiol. Biochem..

[95] Li Y., Niu J., Zhang W., Zhang L., Shang E. (2014). Influence of aqueous media on the ROS-mediated toxicity of ZnO nanoparticles toward green fluorescent protein-expressing *Escherichia coli* under UV-365 irradiation. Langmuir.

[96] Singh R., Cheng S., Singh S. (2020). Oxidative stress-mediated genotoxic effect of zinc oxide nanoparticles on *Deinococcus radiodurans*. 3 Biotech.

[97] El-Shetehy M., Moradi A., Maceroni M., Reinhardt D., Petri-Fink A., Rothen-Rutishauser B., Mauch F., Schwab F. (2021). Silica nanoparticles enhance disease resistance in Arabidopsis plants. Nat. Nanotechnol..

[98] Malandrakis A.A., Kavroulakis N., Chrysikopoulos C.V. (2019). Use of copper, silver and zinc nanoparticles against foliar and soil-borne plant pathogens. Sci. Total Environ..

[99] Burke D.J., Pietrasiak N., Situ S.F., Abenojar E.C., Porche M., Kraj P., Lakliang Y., Samia A.C.S. (2015). Iron oxide and titanium dioxide nanoparticle effects on plant performance and root associated microbes. Int. J. Mol. Sci..

[100] Jat H.S., Datta A., Choudhary M., Sharma P.C., Dixit B., Jat M.L. (2021). Soil enzymes activity: Effect of climate smart agriculture on rhizosphere and bulk soil under cereal based systems of north-west India. Eur. J. Soil Biol..

[101] Chai H., Yao J., Sun J., Zhang C., Liu W., Zhu M., Ceccanti B. (2015). The effect of metal oxide nanoparticles on functional bacteria and metabolic profiles in agricultural soil. Bull. Environ. Contam. Toxicol..

[102] Khati P., Sharma A., Chaudhary P., Singh A.K., Gangola S., Kumar R. (2019). High-throughput sequencing approach to access the impact of nanozeolite treatment on species richness and evenness of soil metagenome. Biocatal. Agric. Biotechnol..

[103] Kukreti B., Sharma A., Chaudhary P., Agri U., Maithani D. (2020). Influence of nanosilicon dioxide along with bioinoculants on Zea mays and its rhizospheric soil. 3 Biotech.

[104] Kumari S., Sharma A., Chaudhary P., Khati P. (2020). Management of plant vigor and soil health using two agriusable nanocompounds and plant growth promotory rhizobacteria in Fenugreek. 3 Biotech.

[105] Chaudhary P., Chaudhary A., Parveen H., Rani A., Kumar G., Kumar R., Sharma A. (2021). Impact of nanophos in agriculture to improve functional bacterial community and crop productivity. BMC Plant Biol..

[106] Agri U., Chaudhary P., Sharma A., Kukreti B. (2022). Physiological response of maize plants and its rhizospheric microbiome under the influence of potential bioinoculants and nanochitosan. Plant Soil.

[107] Chaudhary P., Sharma A., Chaudhary A., Khati P., Gangola S., Maithani D. (2021). Illumina based high throughput analysis of microbial diversity of maize rhizosphere treated with nanocompounds and Bacillus sp.. Appl. Soil Ecol..

[108] Khati P., Parul, Bhatt P., Nisha, Kumar R., Sharma A. (2018). Effect of nanozeolite and plant growth promoting rhizobacteria on maize. 3 Biotech.

[109] Merinero M., Alcudia A., Begines B., Martínez G., Martín-Valero M.J., Pérez-Romero J.A., Mateos-Naranjo E., Redondo-Gómez S., Navarro-Torre S., Torres Y. (2022). Assessing the biofortification of wheat plants by combining a plant growth-promoting rhizobacterium (PGPR) and polymeric Fe-nanoparticles: Allies or enemies?. Agronomy.

[110] Shcherbakova E., Shcherbakov A., Andronov E., Gonchar L., Kalenskaya S., Chebotar V. (2017). Combined pre-seed treatment with microbial inoculants and Mo nanoparticles changes composition of root exudates and rhizosphere microbiome structure of chickpea (*Cicer arietinum* L.) plants. Symbiosis.

[111] Liu R., Lal R. (2015). Potentials of engineered nanoparticles as fertilizers for increasing agronomic productions. Sci. Total Environ..

[112] Mani P.K., Mondal S. (2016). Agri-nanotechniques for plant availability of nutrients. Plant Nanotechnolology. Principles and Particles.

[113] Chhipa H. (2017). Nanofertilizers and nanopesticides for agriculture. Environ. Chem. Lett..

[114] Deng C., Wang Y., Cantu J.M., Valdes C., Navarro G., Cota-Ruiz K., Hernandez-Viezcas J.A., Li C., Elmer W.H., Dimkpa C.O. (2022). Soil and foliar exposure of soybean (*Glycine max*) to Cu: Nanoparticle coating-dependent plant responses. NanoImpact.

[115] Dimkpa C.O., Deng C., Wang Y., Adisa I.O., Zhou J., White J.C. (2023). Chitosan and Zinc Oxide Nanoparticle-Enhanced Tripolyphosphate Modulate Phosphorus Leaching in Soil. ACS Agric. Sci. Technol..

[116] Chen S., Pan Z., Zhao W., Zhou Y., Rui Y., Jiang C., Wang Y., White J.C., Zhao L. (2023). Engineering Climate-Resilient Rice Using a Nanobiostimulant-Based “Stress Training” Strategy. ACS Nano.

[117] Al-Juthery H.W., Lahmod N.R., Al-Taee R.A. (2021). Intelligent, nano-fertilizers: A new technology for improvement nutrient use efficiency (article review). IOP Conf. Ser. Earth Environ. Sci..

[118] Meghana K., Wahiduzzaman M., Vamsi G. (2021). Nanofertilizers in agriculture. Acta Sci. Agric..

[119] Kottegoda N., Sandaruwan C., Priyadarshana G., Siriwardhana A., Rathnayake U.A., Berugoda Arachchige D.M., Kumarasinghe A.R., Dahanayake D., Karunaratne V., Amaratunga G.A. (2017). Urea-hydroxyapatite nanohybrids for slow release of nitrogen. ACS Nano.

[120] Manikandan A., Subramanian K. (2016). Evaluation of zeolite based nitrogen nano-fertilizers on maize growth, yield and quality on inceptisols and alfisols. Int. J. Plant Soil Sci..

[121] Tarafdar J., Raliya R., Mahawar H., Rathore I. (2014). Development of zinc nanofertilizer to enhance crop production in pearl millet (*Pennisetum americanum*). Agric. Res..

[122] Abdel-Aziz H.M., Hasaneen M.N., Omer A.M. (2016). Nano chitosan-NPK fertilizer enhances the growth and productivity of wheat plants grown in sandy soil. Span. J. Agric. Res..

[123] Raliya R., Tarafdar J.C., Biswas P. (2016). Enhancing the mobilization of native phosphorus in the mung bean rhizosphere using ZnO nanoparticles synthesized by soil fungi. J. Agric. Food Chem..

[124] Venkatachalam P., Priyanka N., Manikandan K., Ganeshbabu I., Indiraarulselvi P., Geetha N., Muralikrishna K., Bhattacharya R., Tiwari M., Sharma N. (2017). Enhanced plant growth promoting role of phycomolecules coated zinc oxide nanoparticles with P supplementation in cotton (*Gossypium hirsutum* L.). Plant Physiol. Biochem..

[125] Rui M., Ma C., Hao Y., Guo J., Rui Y., Tang X., Zhao Q., Fan X., Zhang Z., Hou T. (2016). Iron oxide nanoparticles as a potential iron fertilizer for peanut (*Arachis hypogaea*). Front. Plant Sci..

[126] Sadak M.S. (2019). Impact of silver nanoparticles on plant growth, some biochemical aspects, and yield of fenugreek plant (*Trigonella foenum-graecum*). Bull. Natl. Res. Cent..

[127] Wang Y., Jiang F., Ma C., Rui Y., Tsang D.C., Xing B. (2019). Effect of metal oxide nanoparticles on amino acids in wheat grains (*Triticum aestivum*) in a life cycle study. J. Environ. Manag..

[128] Dhlamini B., Paumo H.K., Katata-Seru L., Kutu F.R. (2020). Sulphate-supplemented NPK nanofertilizer and its effect on maize growth. Mater. Res. Express.

[129] Fouda M.M., Abdelsalam N.R., El-Naggar M.E., Zaitoun A.F., Salim B.M., Bin-Jumah M., Allam A.A., Abo-Marzoka S.A., Kandil E.E. (2020). Impact of high throughput green synthesized silver nanoparticles on agronomic traits of onion. Int. J. Biol. Macromol..

[130] Raiesi-Ardali T., Ma’mani L., Chorom M., Moezzi A. (2022). Improved iron use efficiency in tomato using organically coated iron oxide nanoparticles as efficient bioavailable Fe sources. Chem. Biol. Technol. Agric..

[131] Murgueitio-Herrera E., Falconí C.E., Cumbal L., Gómez J., Yanchatipán K., Tapia A., Martínez K., Sinde-Gonzalez I., Toulkeridis T. (2022). Synthesis of iron, zinc, and manganese nanofertilizers, using Andean blueberry extract, and their effect in the growth of cabbage and lupin plants. Nanomaterials.

[132] Liu R., Zhang H., Lal R. (2016). Effects of stabilized nanoparticles of copper, zinc, manganese, and iron oxides in low concentrations on lettuce (*Lactuca sativa*) seed germination: Nanotoxicants or nanonutrients?. Water Air Soil Pollut..

[133] Li J., Hu J., Ma C., Wang Y., Wu C., Huang J., Xing B. (2016). Uptake, translocation and physiological effects of magnetic iron oxide (γ-Fe_2_O_3_) nanoparticles in corn (*Zea mays* L.). Chemosphere.

[134] Subbaiah L.V., Prasad T.N.V.K.V., Krishna T.G., Sudhakar P., Reddy B.R., Pradeep T. (2016). Novel effects of nanoparticulate delivery of zinc on growth, productivity, and zinc biofortification in maize (*Zea mays* L.). J. Agric. Food Chem..

[135] Pradhan S., Patra P., Das S., Chandra S., Mitra S., Dey K.K., Akbar S., Palit P., Goswami A. (2013). Photochemical modulation of biosafe manganese nanoparticles on *Vigna radiata*: A detailed molecular, biochemical, and biophysical study. Environ. Sci. Technol..

[136] Saffan M.M., Koriem M.A., El-Henawy A., El-Mahdy S., El-Ramady H., Elbehiry F., Omara A.E.-D., Bayoumi Y., Badgar K., Prokisch J. (2022). Sustainable production of tomato plants (*Solanum lycopersicum* L.) under low-quality irrigation water as affected by bio-nanofertilizers of selenium and copper. Sustainability.

[137] Sharma A., Kumar S., Singh R. (2022). Synthesis and characterization of a novel slow-release nanourea/chitosan nanocomposite and its effect on *Vigna radiata* L.. Environ. Sci. Nano.

[138] Salama A.M., El-Halim A.E.-H.A.A., Ibrahim M.M., Aiad M.A., El-Shal R.M. (2022). Amendment with nanoparticulate gypsum enhances spinach growth in saline-sodic soil. J. Soil Sci. Plant Nutr..

[139] Abou-Sreea A.I.B., Kamal M., El Sowfy D.M., Rady M.M., Mohamed G.F., Al-Dhumri S.A., Al-Harbi M.S., Abdou N.M. (2022). Small-sized nanophosphorus has a positive impact on the performance of fenugreek plants under soil-water deficit stress: A case study under field conditions. Biology.

[140] Hosseinpour A., Ilhan E., Özkan G., Öztürk H.I., Haliloglu K., Cinisli K.T. (2022). Plant growth-promoting bacteria (PGPBs) and copper (II) oxide (CuO) nanoparticle ameliorates DNA damage and DNA Methylation in wheat (*Triticum aestivum* L.) exposed to NaCl stress. J. Plant Biochem. Biotechnol..

[141] Van Dijk M., Morley T., Rau M.L., Saghai Y. (2021). A meta-analysis of projected global food demand and population at risk of hunger for the period 2010–2050. Nat. Food.

[142] Matny O. (2015). Fusarium head blight and crown rot on wheat & barley: Losses and health risks. Adv. Plants Agric. Res..

[143] Khan M.R., Sharma R.K. (2020). Fusarium-nematode wilt disease complexes, etiology and mechanism of development. Indian Phytopathol..

[144] Worrall E.A., Hamid A., Mody K.T., Mitter N., Pappu H.R. (2018). Nanotechnology for plant disease management. Agronomy.

[145] Elmer W., White J.C. (2018). The future of nanotechnology in plant pathology. Annu. Rev. Phytopathol..

[146] Mittal D., Kaur G., Singh P., Yadav K., Ali S.A. (2020). Nanoparticle-based sustainable agriculture and food science: Recent advances and future outlook. Front. Nanotechnol..

[147] Servin A., Elmer W., Mukherjee A., De la Torre-Roche R., Hamdi H., White J.C., Bindraban P., Dimkpa C. (2015). A review of the use of engineered nanomaterials to suppress plant disease and enhance crop yield. J. Nanopart. Res..

[148] Wang Y., Deng C., Shen Y., Borgatta J., Dimkpa C.O., Xing B., Dhankher O.P., Wang Z., White J.C., Elmer W.H. (2022). Surface coated sulfur nanoparticles suppress fusarium disease in field grown tomato: Increased yield and nutrient biofortification. J. Agric. Food Chem..

[149] Okey-Onyesolu C.F., Hassanisaadi M., Bilal M., Barani M., Rahdar A., Iqbal J., Kyzas G.Z. (2021). Nanomaterials as nanofertilizers and nanopesticides: An overview. ChemistrySelect.

[150] Park H.J., Kim S.H., Kim H.J., Choi S.H. (2006). A new composition of nanosized silica-silver for control of various plant diseases. Plant Pathol. J..

[151] Ponmurugan P., Manjukarunambika K., Elango V., Gnanamangai B.M. (2016). Antifungal activity of biosynthesised copper nanoparticles evaluated against red root-rot disease in tea plants. J. Exp. Nanosci..

[152] Elbasuney S., El-Sayyad G.S., Attia M.S., Abdelaziz A.M. (2022). Ferric oxide colloid: Towards green nano-fertilizer for tomato plant with enhanced vegetative growth and immune response against fusarium wilt disease. J. Inorg. Organomet. Polym. Mater..

[153] El-Sherif A.G., Gad S.B., Megahed A.A., Sergany M.I. (2019). Induction of tomato plants resistance *to Meloidogyne incognita* infection by mineral and nano-fertilizer. J. Entomol. Nematol..

[154] Prom-U-Thai C., Rashid A., Ram H., Zou C., Guilherme L.R.G., Corguinha A.P.B., Guo S., Kaur C., Naeem A., Yamuangmorn S. (2020). Simultaneous biofortification of rice with zinc, iodine, iron and selenium through foliar treatment of a micronutrient cocktail in five countries. Front. Plant Sci..

[155] Wang Y., Chen S., Deng C., Shi X., Cota-Ruiz K., White J.C., Zhao L., Gardea-Torresdey J.L. (2021). Metabolomic analysis reveals dose-dependent alteration of maize (*Zea mays* L.) metabolites and mineral nutrient profiles upon exposure to zerovalent iron nanoparticles. NanoImpact.

[156] Almendros P., González D., Fernández M.D., García-Gomez C., Obrador A. (2022). Both Zn biofortification and nutrient distribution pattern in cherry tomato plants are influenced by the application of ZnO nanofertilizer. Heliyon.

[157] Rahman M.H., Hasan M.N., Nigar S., Ma F., Aly Saad Aly M., Khan M.Z.H. (2021). Synthesis and characterization of a mixed nanofertilizer influencing the nutrient use efficiency, productivity, and nutritive value of tomato fruits. ACS Omega.

[158] Abou-yuoseff A., El–Khair A., El–Mohtasem M., Shawer S. (2022). Impacts of Nano-Fertilizers and Chemical Fertilizers on Plant Growth and Nutrient Uptake by Faba Bean (*Vicia faba* L.) Plant. Al-Azhar J. Agric. Res..

[159] Dimkpa C.O., Campos M.G., Fugice J., Glass K., Ozcan A., Huang Z., Singh U., Santra S. (2022). Synthesis and characterization of novel dual-capped Zn–urea nanofertilizers and application in nutrient delivery in wheat. Environ. Sci. Adv..

[160] Rajonee A.A., Zaman S., Huq S.M.I. (2017). Preparation, characterization and evaluation of efficacy of phosphorus and potassium incorporated nano fertilizer. Adv. Nanopart..

[161] Dhansil A., Zalawadia N., Prajapat B.S., Yadav K. (2018). Effect of nano phosphatic fertilizer on nutrient content and uptake by pearl millet (*Pennisetum glaucum* L.) crop. Int. J. Curr. Microbiol. Appl. Sci.

[162] Kalwani M., Chakdar H., Srivastava A., Pabbi S., Shukla P. (2022). Effects of nanofertilizers on soil and plant-associated microbial communities: Emerging trends and perspectives. Chemosphere.

[163] Rajput V., Minkina T., Suskova S., Mandzhieva S., Tsitsuashvili V., Chapligin V., Fedorenko A. (2018). Effects of copper nanoparticles (CuO NPs) on crop plants: A mini review. BioNanoScience.

[164] Ameen F., Alsamhary K., Alabdullatif J.A., ALNadhari S. (2021). A review on metal-based nanoparticles and their toxicity to beneficial soil bacteria and fungi. Ecotoxicol. Environ. Saf..

[165] Grün A.-L., Manz W., Kohl Y.L., Meier F., Straskraba S., Jost C., Drexel R., Emmerling C. (2019). Impact of silver nanoparticles (AgNP) on soil microbial community depending on functionalization, concentration, exposure time, and soil texture. Environ. Sci. Eur..

[166] McGee C., Storey S., Clipson N., Doyle E. (2017). Soil microbial community responses to contamination with silver, aluminium oxide and silicon dioxide nanoparticles. Ecotoxicology.

[167] Juan W., Kunhui S., Zhang L., Youbin S. (2017). Effects of silver nanoparticles on soil microbial communities and bacterial nitrification in suburban vegetable soils. Pedosphere.

[168] Cota-Ruiz K., Ye Y., Valdes C., Deng C., Wang Y., Hernández-Viezcas J.A., Duarte-Gardea M., Gardea-Torresdey J.L. (2020). Copper nanowires as nanofertilizers for alfalfa plants: Understanding nano-bio systems interactions from microbial genomics, plant molecular responses and spectroscopic studies. Sci. Total Environ..

[169] Parada J., Rubilar O., Diez M., Cea M., Da Silva A.S.A., Rodríguez-Rodríguez C.E., Tortella G. (2019). Combined pollution of copper nanoparticles and atrazine in soil: Effects on dissipation of the pesticide and on microbiological community profiles. J. Hazard. Mater..

[170] You T., Liu D., Chen J., Yang Z., Dou R., Gao X., Wang L. (2018). Effects of metal oxide nanoparticles on soil enzyme activities and bacterial communities in two different soil types. J. Soils Sediments.

[171] Gupta A., Bano A., Rai S., Pathak N., Sharma S. (2020). New insights into application of nanoparticles for plant growth promotion: Present and future prospects. Biog. Nano-Part. Their Use Agro-Ecosyst..

[172] Eliaspour S., Seyed Sharifi R., Shirkhani A., Farzaneh S. (2020). Effects of biofertilizers and iron nano-oxide on maize yield and physiological properties under optimal irrigation and drought stress conditions. Food Sci. Nutr..

[173] Pudake R.N., Chauhan N., Kole C. (2019). Nanoscience for Sustainable Agriculture.

[174] Gahoi P., Omar R.A., Verma N., Gupta G.S. (2021). Rhizobacteria and Acylated homoserine lactone-based nanobiofertilizer to improve growth and pathogen defense in *Cicer arietinum* and *Triticum aestivum* Plants. ACS Agric. Sci. Technol..

[175] Davod T., Reza Z., Ali V.A., Mehrdad C. (2011). Effects of nanosilver and nitroxin biofertilizer on yield and yield components of potato minitubers. Int. J. Agric. Biol.

[176] Mardalipour M., Zahedi H., Sharghi Y. (2014). Evaluation of nano biofertilizer efficiency on agronomic traits of spring wheat at different sowing date. Biol. Forum Int. J. Res. Trend.

[177] Farnia A., Omidi M.M., Farnia A. (2015). Effect of nano-zinc chelate and nano-biofertilizer on yield and yield components of maize (*Zea mays* L.), under water stress condition. Indian J. Nat. Sci..

[178] Mir S., Sirousmehr A., Shirmohammadi E. (2015). Effect of nano and biological fertilizers on carbohydrate and chlorophyll content of forage sorghum (*Speedfeed hybrid*). Int. J. Biosci..

[179] Gatahi D., Wanyika H., Kihurani A., Ateka E., Kavoo A. Use of bio-nanocomposites in enhancing bacterial wilt plant resistance, tomato production and water conservation in greenhouse farming. Proceedings of the 10th JKUAT Scientific, Technological and Industrialization Conference.

[180] Nawaz S., Bano A. (2020). Effects of PGPR (*Pseudomonas* sp.) and Ag-nanoparticles on enzymatic activity and physiology of cucumber. Recent Pat. Food Nutr. Agric..

[181] Jahangir S., Javed K., Bano A. (2020). Nanoparticles and plant growth promoting rhizobacteria (PGPR) modulate the physiology of onion plant under salt stress. Pak. J. Bot..

[182] Kulkarni N., Muddapur U. (2014). Biosynthesis of metal nanoparticles: A review. J. Nanotechnol..

[183] Golbashy M., Sabahi H., Allahdadi I., Nazokdast H., Hosseini M. (2017). Synthesis of highly intercalated urea-clay nanocomposite via domestic montmorillonite as eco-friendly slow-release fertilizer. Arch. Agron. Soil Sci..

[184] Vejan P., Abdullah R., Khadiran T., Ismail S., Nasrulhaq Boyce A. (2016). Role of plant growth promoting rhizobacteria in agricultural sustainability—A review. Molecules.

[185] Moradipour M., Saberi-Riseh R., Mohammadinejad R., Hosseini A. (2019). Nano-encapsulation of plant growth-promoting rhizobacteria and their metabolites using alginate-silica nanoparticles and carbon nanotube improves UCB1 pistachio micropropagation. J. Microbiol. Biotechnol..

[186] Moradi Pour M., Saberi Riseh R., Skorik Y.A. (2022). Sodium alginate–gelatin nanoformulations for encapsulation of *Bacillus velezensis* and their use for biological control of pistachio gummosis. Materials.

[187] Saberi Riseh R., Moradi Pour M., Ait Barka E. (2022). A Novel route for double-layered encapsulation of *Streptomyces fulvissimus* Uts22 by alginate–Arabic gum for controlling of *Pythium aphanidermatum* in Cucumber. Agronomy.

[188] Panichikkal J., Prathap G., Nair R.A., Krishnankutty R.E. (2021). Evaluation of plant probiotic performance of *Pseudomonas* sp. encapsulated in alginate supplemented with salicylic acid and zinc oxide nanoparticles. Int. J. Biol. Macromol..

[189] Saberi-Rise R., Moradi-Pour M. (2020). The effect of Bacillus subtilis Vru1 encapsulated in alginate–bentonite coating enriched with titanium nanoparticles against *Rhizoctonia solani* on bean. Int. J. Biol. Macromol..

[190] Moradi Pour M., Saberi Riseh R., Ranjbar-Karimi R., Hassanisaadi M., Rahdar A., Baino F. (2022). Microencapsulation of *Bacillus velezensis* using alginate-gum polymers enriched with TiO_2_ and SiO_2_ nanoparticles. Micromachines.

